# Decoding Urinary Tract Infection Trends: A 5-Year Snapshot from Central Portugal

**DOI:** 10.3390/clinpract15010014

**Published:** 2025-01-06

**Authors:** Francisco Rodrigues, Patrícia Coelho, Sónia Mateus, Armando Caseiro, Hatem Eideh, Teresa Gonçalves, Miguel Castelo Branco

**Affiliations:** 1 Polytechnic Institute of Castelo Branco, Dr. Lopes Dias Higher Health School, 6000-084 Castelo Branco, Portugal; patriciacoelho@ipcb.pt (P.C.); soniamateus@ipcb.pt (S.M.); 2 Sport Physical Activity and Health Research & Innovation Center (Sprint), Polytechnic Institute of Castelo Branco, 6000-084 Castelo Branco, Portugal; 3 Coimbra Health School, Polytechnic University of Coimbra, 3045-093 Coimbra, Portugal; armandocaseiro@estesc.ipc.pt; 4 H&TRC—Health & Technology Research Center, Coimbra Health School, Polytechnic University of Coimbra, Rua 5 de Outubro, 3045-043 Coimbra, Portugal; 5 Faculty of Sport Science and Physical Education, University of Coimbra, CIDAF, 3000-456 Coimbra, Portugal; 6 Department of Medical Laboratory Sciences, Al-Quds University, Abu Dis P144, Palestine; heideh@staff.alquds.edu; 7 FMUC—Faculty of Medicine, University of Coimbra, 3004-504 Coimbra, Portugal; tmfog@ci.uc.pt; 8 CNC-UC—Center for Neuroscience and Cell Biology, University of Coimbra, 3004-504 Coimbra, Portugal; 9 CIBB—Centre for Innovative Biomedicine and Biotechnology, University of Coimbra, 3004-504 Coimbra, Portugal; 10 Faculty of Health Sciences, University of Beira Interior, 6201-001 Covilha, Portugal; mcbranco@fcsaude.ubi.pt

**Keywords:** urinary tract infections (UTIs), antibiotic resistance, antibiotic stewardship, epidemiology of UTIs, antimicrobial consumption, Portugal

## Abstract

Introduction: This study analyzes urinary tract infections (UTIs) in a hospital in Central Portugal over a five-year period, focusing on bacterial prevalence, patient demographics, and antibiotic resistance patterns. This investigation aims to provide insights that can guide improved infection control and treatment strategies. Methods: A total of 6161 positive urine cultures collected over five years were examined, with particular emphasis on 2019 due to a peak in infection rates. The analysis explored bacterial prevalence, demographic factors such as sex and clinical service origin, and antibiotic resistance. Special attention was given to hospitalized patients, especially those undergoing invasive procedures, due to their increased vulnerability to infection. Results: This study found that UTIs were more prevalent in female patients, reflecting anatomical susceptibilities. Hospitalized individuals, particularly those requiring invasive procedures, were at greater risk. The predominant bacteria were *Escherichia coli*, *Klebsiella pneumoniae*, and *Enterococcus faecalis*, with differences in prevalence by patient sex and service origin. Resistance to Imipenem in *E. coli* increased, raising concerns about last-resort treatments. However, resistance to other antibiotics declined, suggesting improvements due to recent stewardship measures. During the COVID-19 pandemic, overall antibiotic consumption decreased due to changes in clinical practices. Conclusion: The findings highlight the importance of strict infection control, targeted prevention measures, and rational antibiotic use to combat resistance. Ongoing surveillance and personalized treatment approaches are essential to improve UTI management and outcomes.

## 1. Introduction

Urinary tract infections (UTIs) are among the most common infections in humans, currently ranking as the second most prevalent infection worldwide. Their incidence is fairly consistent across European countries but is notably higher in African nations with lower socioeconomic conditions [1]. UTIs are characterized by the presence of microorganisms in the kidneys, ureters, bladder, or urethra, with normal microbiota being tolerated in the urethra under typical conditions [2]. Due to anatomical factors, such as the proximity of the vagina and anus and a shorter urethra, females are at a higher risk of UTIs compared to males, alongside a rate of asymptomatic colonization which may range from 1% to 5% of all positive urine cultures. Besides being one of the most common infections in adults, UTIs are among the leading causes of hospitalization in neonates and infants, with a higher prevalence in males, in contrast to the epidemiological data observed in adults [2]. A variety of permanent or temporary conditions can elevate the likelihood of developing UTIs, including genetic factors (e.g., a deficiency in the CXCR1 receptor for interleukin 8, genetically induced kidney damage), diabetes mellitus, pregnancy, early history of UTIs, circumcision status, neurogenic bladder, kidney transplant, menopause, prostatic hypertrophy, catheterization, urinary tract obstructions, and elimination disorders [3,4]. The recurrence of UTIs is closely linked to the fact that the urinary system has a direct connection to the external environment via the urethra [5].

UTIs can arise through several routes: the hematogenous route, which is more common in neonates and associated with urosepsis; the lymphatic route, rare and typically linked to severe intestinal infections or retroperitoneal abscesses; and, predominantly, the ascending route, which accounts for the majority of UTIs in humans. Fortunately, the body has defense mechanisms such as urination, the natural microbiota of the distal urethra, specific urine composition (low osmolality, high urea, and organic acid concentration), and the anti-inflammatory and antimicrobial properties of the bladder mucosa, in addition to the immune system [6,7,8,9]. Among the preventive strategies, the consumption of red fruits, such as cranberries, stands out, as they have been associated with a reduced risk of UTIs due to the presence of proanthocyanins, which can inhibit bacterial adhesion to the urinary epithelium.

Early diagnosis is essential to minimize the risk of complicated UTIs, which can lead to kidney damage and subsequent scarring. An initial UTI diagnosis typically involves a combination of clinical symptoms (burning or pain during urination, frequent urination) and a positive urine summary (type II urine), supplemented by microbiological testing to identify the strain and determine its antibiotic susceptibility profile. Additional clinical tests, such as blood count and C-reactive protein levels, may offer further signs of infection, although more specific markers like procalcitonin or blood cultures have not yet proven to be decisive at early diagnostic stages [10,11,12].

Globally, *Escherichia coli* (*E. coli*) is the most prevalent bacteria in community-acquired UTIs, followed by *Staphylococcus saprophyticus*, *Proteus* species, *Klebsiella*, and *Enterococcus faecalis* (*E. faecalis*). In hospital settings, while *E. coli* remains predominant, there has been a noted increase in other strains, including *Proteus* species., *Pseudomonas aeruginosa*, *Klebsiella*, and some fungi, notably *Candida* species [13,14]. Hospital-acquired UTIs often include an increase in less common bacterial species, such as those of the genus *Proteus* species, compared to community-acquired infections [6,10,15]. Although PCR (polymerase chain reaction) technology is becoming more widespread, traditional microbiological tests often require 24 to 48 h to yield definitive results. Although the ideal diagnosis involves the precise identification of the pathogen and its resistance profile, urgent clinical situations may require empirical therapy based on local epidemiological data [16,17].

This study aims to contribute to that body of knowledge by examining the sociodemographic characteristics of patients with positive urine cultures from 2018 to 2022, analyzing the main strains identified during this period, and evaluating the response of these strains to the tested antibiotics.

## 2. Materials and Methods

A retrospective observational study was conducted on all positive urine cultures obtained from January 2018 to December 2022 at a hospital center in Central Portugal, totaling 6161 samples. Data collection was supported by a computerized system and included variables such as gender (male or female), age, patient origin (emergency, internment, external consult, or day hospital), the isolated bacterial strain, and antibiotic susceptibility (sensitive or resistant). The origin of the patients reflects the scope of the analyzed hospital service, including emergency care, inpatient admissions, and outpatient consultations. Government databases on antibiotic consumption in Portugal by year and institution were also consulted. These data were compiled into a database for subsequent statistical analysis. IBM SPSS Statistics, version 29.0.1 for Mac iOS, was used for data processing, employing descriptive statistical methods based on the study’s objectives. This research was approved by the Ethics Committee and the Data Protection Officer of the University of Beira Interior, with all ethical guidelines strictly followed. Due to the retrospective design and the absence of any patient-identifying information, informed consent was waived. This study was part of the ITUCIP project (Urinary Tract Infections in the Central Interior of Portugal).

## 3. Results

**Figure 1:**
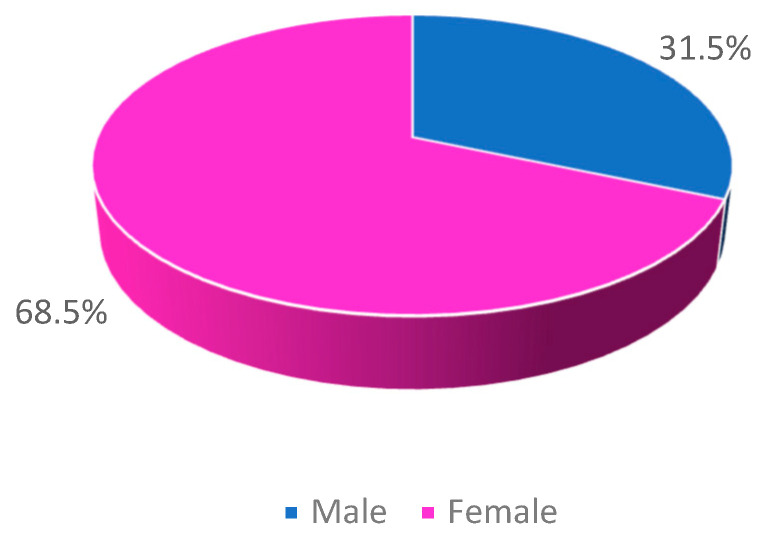
Sample distribution by sex.

A total of 6161 urine samples positive for the presence of bacteria were analyzed, collected between the years 2018 and 2022. Most samples were from the year 2019 (22.80%), followed by 2018 (21.40%) and 2021 (19.80%). Female sex was the most prevalent, as shown in Figure 1.

Analyzing the origin of the strains by service, it was observed that most came from internment (47.5%), followed by external consult (35.3%), emergency care (16.6%), and day hospital (0.6%).

**Figure 2:**
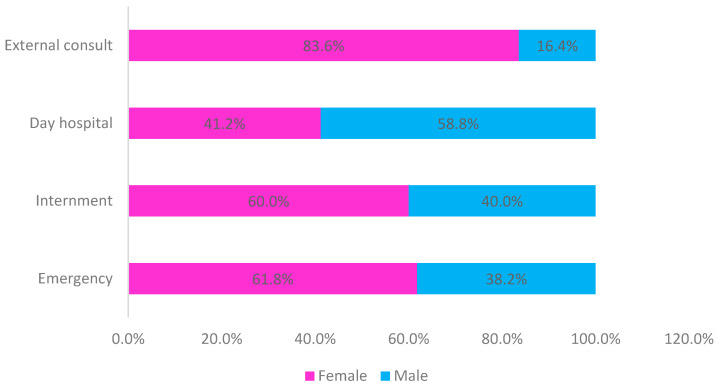
Sample distribution by place of origin and sex (*n* = 6161).

Men had a higher prevalence only in samples from the day hospital, while, in all other locations, samples were predominantly from women (Figure 2).

**Figure 3:**
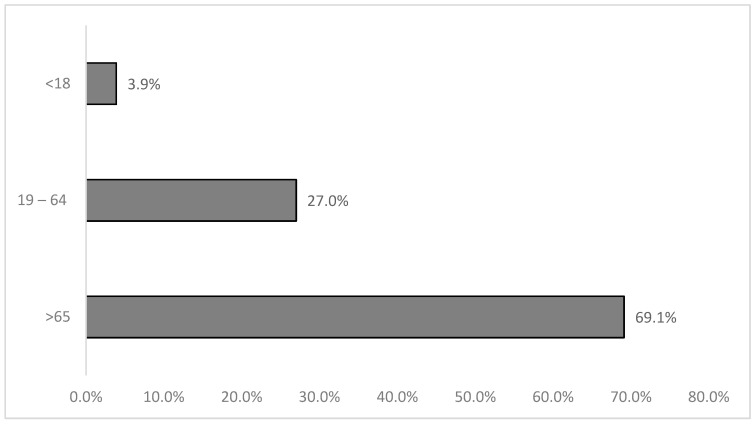
Sample distribution by age group (*n* = 6161).

Regarding age, the vast majority were over 65 years old (Figure 3). Although the mean and standard deviation were not available, the age range of patients ranged from 1 to 97 years.

**Figure 4:**
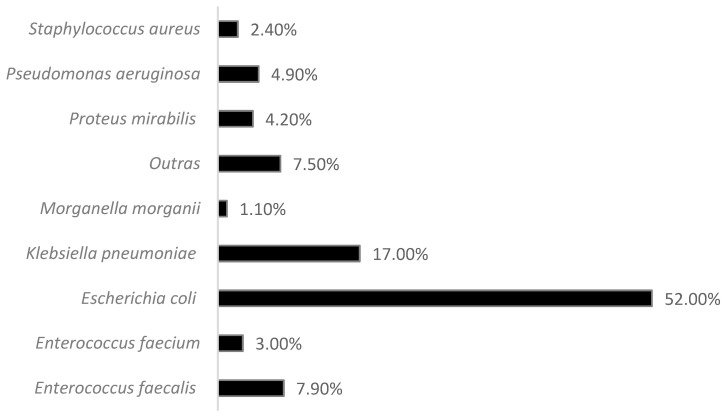
Sample distribution by strains (*n* = 6161).

Analyzing the most prevalent strains, *Escherichia coli* was predominant, followed by *Klebsiella pneumoniae* and *Enterococcus faecalis* (Figure 4). 

**Figure 5:**
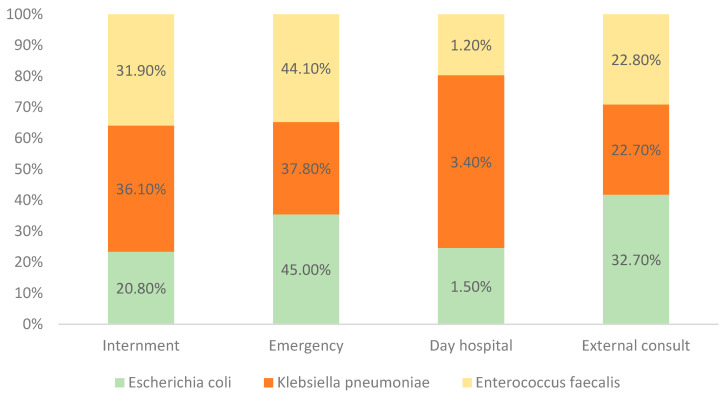
Distribution of the most prevalent strains by place of origin (*n* = 4738).

Analyzing the three main strains found, we observed that most *E. coli* cases originated from patients after external consult, while the majority of *Klebsiella pneumoniae* and *Enterococcus faecalis* cases came from internment (Figure 5).

**Figure 6:**
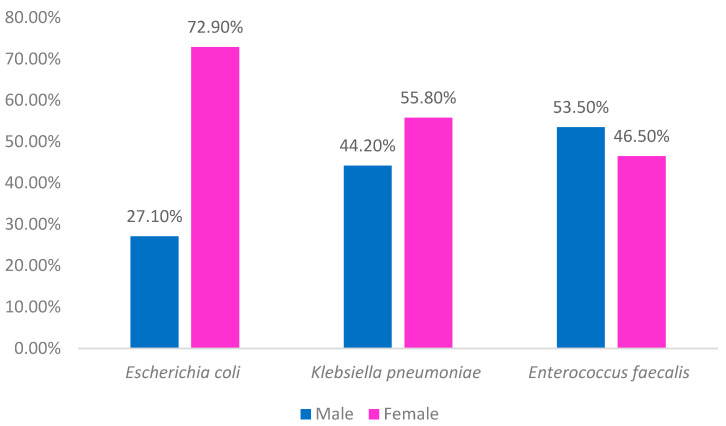
Distribution of the most prevalent strains by sex (*n* = 4738).

Observing the three strains by gender, it was noted that most *E. coli* and *Klebsiella pneumoniae* cases had been identified in female individuals, while the majority of *Enterococcus faecalis* cases were from male individuals (Figure 6).

Analyzing the behavior of the two most prevalent bacteria against the antibiotics tested in the Health Unit, specifically the percentage of resistant strains per year, we observed that *Escherichia coli* increased its resistance to one antibiotic (Imipenem) between 2018 and 2022, decreased its resistance to fifteen antibiotics (Amikacin, Ampicillin, Amoxicillin/Clavulanic Acid, Cefepime, Ceftazidime, Cefotaxime, Cefuroxime, Cefuroxime Axetil, Ciprofloxacin, Fosfomycin, Gentamicin, Meropenem, Piperacillin/Tazobactam, High-Level Streptomycin, and Trimethoprim/Sulfamethoxazole), and remained stable against two antibiotics (Colistin and High-Level Gentamicin). *Klebsiella pneumoniae* showed increased resistance between 2018 and 2022 to five antibiotics (Ceftazidime, Cefotaxime, Cefuroxime, Cefuroxime Axetil, and Fosfomycin), decreased resistance to eight antibiotics (Amikacin, Amoxicillin/Clavulanic Acid, Cefepime, Ertapenem, Gentamicin, Meropenem, Piperacillin/Tazobactam, and Trimethoprim/Sulfamethoxazole), and remained stable against two antibiotics (Colistin and Ciprofloxacin) (Table 1 and Table 2).

**Figure 7:**
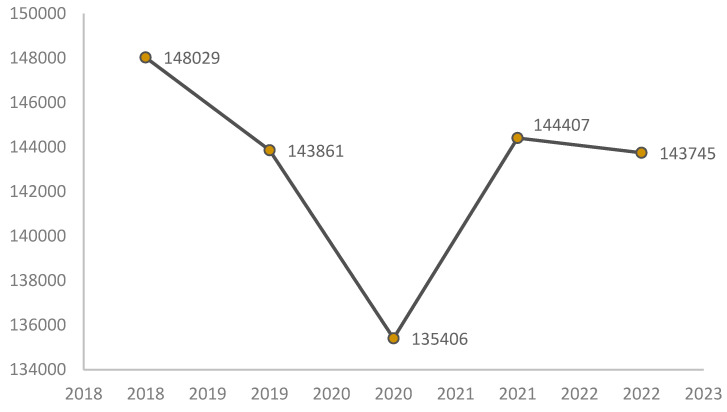
Antibiotic units consumed in the health institution.

The antibiotic consumption in this Health Unit between 2018 and 2022 was also analyzed, as shown in Figure 7, with 2020 being, once again, the year of lowest consumption. The 2022 values showed a slight decrease compared to 2021, not yet sufficient to indicate a clear trend (Figure 7). This consumption concerned all antibiotics consumed in the Health Unit, for all pathologies. It was not possible to perform an analysis by type of antibiotic based on the data provided.

## 4. Discussion

### 4.1. Analysis of Samples and Prevalence by Year

During the study period, 6161 positive urine samples were identified, with infection rates peaking in 2019, alongside a notable predominance in female patients. This finding is in line with the existing literature, which identifies anatomical factors, such as the shorter urethra in women and the proximity of the urethra to the anus, as contributors to their higher susceptibility to urinary tract infections (UTIs) [18,19]. Furthermore, the drop in UTI cases observed in 2021 and 2022 could have been linked to the COVID-19 pandemic, which significantly affected access to healthcare services. Global data during this time show a similar pattern, with many health systems experiencing a strain which limited routine care, while public health efforts prioritized pandemic response [20,21]. Thus, lower UTI case numbers may reflect a mix of reduced patient access, shifts in healthcare-seeking behavior, and a possible underreporting bias during the pandemic.

### 4.2. Origin of Strains by Service

The majority of positive samples in this study originated from internment, emphasizing the role of hospitalization and invasive medical devices, such as catheters, in increasing UTI risk. This finding aligns with established research that correlates prolonged hospital stays and device use with heightened infection rates, underscoring the critical need for stringent infection control measures within healthcare settings [22,23,24]. Notably, patients undergoing prolonged hospital treatments often require catheters or other interventions that heighten their exposure to pathogens, making UTIs a common healthcare-associated infection. On the other hand, the second-highest source of positive samples came from external consult, highlighting the importance of early detection and timely treatment in non-hospitalized patients. Effective external consult, with timely diagnosis and management, can prevent complications and improve clinical outcomes, stressing the need for accessible UTI diagnostic and treatment services in primary care settings [25,26].

### 4.3. Prevalence by Gender

The data reveal that *E. coli* and *Klebsiella pneumoniae* infections were predominantly found in women, while *Enterococcus faecalis* infections were more common in men. Biological and social factors may explain this gender-based difference. Previous studies show that male patients tend to present with *Enterococcus faecalis*-related UTIs more frequently, which may be associated with predisposing conditions like benign prostatic hyperplasia [27]. In contrast, women generally seek medical attention earlier in the course of UTI symptoms, while men often present with complications or infections associated with other underlying health conditions. The observed gender discrepancy aligns with prior studies, indicating that physiological differences, health-seeking behaviors, and comorbid conditions influence UTI patterns across genders [28]. In urinary infections, there is always a very high prevalence of female cases, although, in this specific geographical area, there was also a prevalence of women compared to men in the general population, but this does not seem to have had an influence on the results obtained, as this predominance has not been demonstrated in other pathologies [29]. These findings suggest the need for differentiated diagnostic and therapeutic approaches that consider these gender-specific factors. Recent evidence indicates that personalized and short-duration antibiotic therapies are equally effective in eradicating infections, significantly reducing the risk of bacterial resistance in subsequent infections. The implementation of these strategies is crucial given the reported increase in bacterial resistance [29].

### 4.4. Antibiotic Resistance Behavior

The increasing resistance of *E. coli* to Imipenem presents a major clinical concern, as Imipenem is widely regarded as a last-line treatment for severe infections. This pattern is reflective of a global trend, with studies documenting rising resistance to broad-spectrum antibiotics, driven in part by overuse and misuse [30,31,32]. The decrease in resistance observed in *E. coli* strains to 16 other antibiotics, however, suggests that recent rational antibiotic use policies may be contributing to some success in managing resistance. For Klebsiella pneumoniae, increased resistance to five antibiotics underscores the urgency of implementing and revising antibiotic stewardship protocols in healthcare settings, particularly in hospitals, where selective pressures may accelerate resistance [33]. The contrasting trends in resistance between *E. coli* and *Klebsiella pneumoniae* highlight the complex dynamics of antibiotic efficacy, which require the continuous adaptation of protocols based on current resistance patterns.

### 4.5. Antibiotic Consumption

The data on antibiotic consumption from this Health Unit reflect global usage patterns, as they encompass all antibiotic prescriptions for both inpatients and outpatients. Antibiotic usage decreased in 2020, likely due to the pandemic’s impact on clinical practices, where a greater emphasis was placed on infection prevention and control. Some countries set ambitious targets for reducing antibiotic consumption, with the Norwegian government, for example, aiming for a 30% reduction by 2020—a goal which may have been facilitated by pandemic-related changes in health priorities [34]. While a slight reduction was also noted in 2022 compared to 2021, this decrease is not yet indicative of a lasting trend. It is also important to recognize that, during the pandemic, antibiotic use rose in patients with COVID-19 for treating secondary infections, although the overall prescription rates tended to decrease [35,36].

Maintaining a rational approach to antibiotic prescription remains essential to controlling bacterial resistance. Integrating antibiotic consumption analyses into medical education and patient awareness initiatives could support this goal, promoting conscientious prescription practices. Continuous training on antibiotic stewardship should thus remain a priority, ensuring that healthcare providers and patients alike understand the importance of targeted, rational antibiotic use to preserve antibiotic effectiveness over time [37].

This study did not collect data on predisposing conditions, such as diabetes or pregnancy, which may influence susceptibility to UTIs. Future studies should include these factors for more comprehensive analyses.

Although data on the duration of hospitalization and pre-existing conditions, such as diabetes, could have enriched the analysis, this information was not available in this retrospective study.

## 5. Conclusions

This study provided a comprehensive insight into urinary tract infections at a Hospital Center in Portugal, highlighting the predominance of *Escherichia coli* as the primary pathogenic agent, followed by *Klebsiella pneumoniae* and *Enterococcus faecalis*. The analysis of data collected over five years revealed a high incidence of infections, especially among women, along with a concerning increase in bacterial resistance, particularly to the antibiotic Imipenem. These findings underscore the urgent need to implement effective infection control strategies in hospital settings, where risk factors are heightened.

Additionally, the differences in prevalence between sexes suggest that personalized treatment approaches may be necessary, considering the specific characteristics of men and women in managing UTIs. The observed decrease in antibiotic consumption in 2020, along with a slight decline in 2022, indicates that rational antibiotic use policies may be beginning to take effect. However, it is crucial to continue monitoring bacterial resistance to ensure the effectiveness of the therapies employed.

In summary, this work emphasizes the importance of public health interventions that promote education on the appropriate use of antibiotics and infection prevention. The integration of preventive and therapeutic measures can significantly contribute to reducing infection and resistance rates, thereby improving clinical outcomes for patients.

## Figures and Tables

**Table 1:** *Escherichia coli* resistance to antibiotics between 2018 and 2022.

2018	2019	2020	2021	2022
Amikacina				
4.1%	3%	3.6%	3.3%	3%
Ampicillin				
45.3%	48.5%	49%	46.3%	42.5%
Amoxicilina/Clavulanic Acid				
36.6%	37.7%	38.3%	34.2%	33.5%
Cefepime				
12.2%	9.7%	11.3%	8.6%	4.7%
Ceftazidime				
13.3%	8.9%	11.8%	9.5%	6.5%
Cefotaxime				
14.1%	12.2%	14.1%	11.4%	7.2%
Cefuroxime				
17.9%	19.4%	21.4%	18.8%	11.9%
Cefuroxime Axetil				
17.9%	19.4%	21.4%	18.8%	11.9%
Ciprofloxacin				
23.3%	28.5%	25.9%	19.3%	16%
Colistin				
0%	0%	0%	0%	0%
Ertapenem				
0.4%	0%	0%	0%	0%
Fosfomycin				
3.3%	5.0%	3.2%	2.3%	2.1%
Gentamicin				
13%	9.1%	11.4%	10%	6%
High-Level Gentamicin				
50%	50%	50%	50%	50%
Imipenem				
44.4%	0%	0%	0%	56.7%
Meropenem				
24.3%	0%	0%	0%	0%
Nitrofurantoin				
0.5%	0.8%	0.2%	0.9%	0.5%
Piperacillin/Tazobactam				
9.8%	6.4%	6.7%	3.7%	4.1%
High-Level Streptomycin				
27.3%	20%	25.3%	29%	17.5%
Trimethoprim/Sulfamethoxazole				
28.7%	31.9%	28.3%	26%	24.1%

Legend: green, decreased resistance; and red, increased resistance.

**Table 2:** *Klebsiella pneumoniae* resistance to antibiotics between 2018 and 2022.

2018	2019	2020	2021	2022
Amikacin				
25.5%	11%	24.4%	14.1%	11%
Ampicillin				
100%	100%	100%	100%	100%
Amoxicillin/Clavulanic Acid				
62.7%	62.7%	68.9%	56.3%	62.3%
Cefepime				
51%	48.3%	57%	45.5%	49.7%
Ceftazidime				
53.9%	50.8%	58%	47.4%	55%
Cefotaxime				
52%	50%	57.5%	46.9%	53.9%
Cefuroxime				
53.9%	55.1%	58.5%	49.3%	56%
Cefuroxime Axetyl				
53.9%	55.1%	58.5%	49.3%	56%
Colistin				
1.0%	1.0%	1.0%	1.0%	1.0%
Ciprofloxacin				
46.1%	52.5%	50.3%	43.7%	46.1%
Ertapenem				
26.5%	14.4%	31.6%	23.5%	20.4%
Fosfomycin				
22.5%	21.2%	33.7%	41.3%	33.3%
Gentamicin				
51%	46.6%	46.6%	42.7%	28.8%
Meropen				
59.3%	11%	19.2%	16.4%	11.5%
Piperacillin/Tazobactam				
58.8%	53.8%	63.7%	51.6%	56.3%
Trimethoprim/Sulfamethoxazole				
55.9%	52.5%	54.4%	45.5%	55%

Legend: green, decreased resistance; and red, increased resistance.

## Data Availability

The data forms part of a database that can be made available for consultation, based on plausible justifications. All data is anonymized.
